# Pharmacological Chaperones: A Therapeutic Approach for Diseases Caused by Destabilizing Missense Mutations

**DOI:** 10.3390/ijms21020489

**Published:** 2020-01-13

**Authors:** Ludovica Liguori, Maria Monticelli, Mariateresa Allocca, Bruno Hay Mele, Jan Lukas, Maria Vittoria Cubellis, Giuseppina Andreotti

**Affiliations:** 1 Dipartimento di Scienze e Tecnologie Ambientali, Biologiche e Farmaceutiche, Università degli Studi della Campania “Luigi Vanvitelli”, 81100 Caserta, Italy; lud.liguori@gmail.com (L.L.); mariateresa.allocca@gmail.com (M.A.); 2 Istituto di Chimica Biomolecolare–CNR, 80078 Pozzuoli, Italy; gandreotti@icb.cnr.it; 3 Dipartimento di Biologia, Università Federico II, 80126 Napoli, Italy; maria.monticelli@yahoo.com; 4 Integrative Marine Ecology Department, Stazione Zoologica Anton Dohrn, Villa Comunale, 80121 Naples, Italy; bruno.haymele@unina.it; 5 Translational Neurodegeneration Section “Albrecht-Kossel”, Department of Neurology, University Medical Center Rostock, University of Rostock, 18147 Rostock, Germany; jan.lukas@med.uni-rostock.de; 6 Center for Transdisciplinary Neurosciences Rostock (CTNR), University Medical Center Rostock, University of Rostock, 18147 Rostock, Germany

**Keywords:** disease, pharmacological chaperones, low molecular weight drugs, protein stability, drug repositioning, lysosomal storage disorders

## Abstract

The term “pharmacological chaperone” was introduced 20 years ago. Since then the approach with this type of drug has been proposed for several diseases, lysosomal storage disorders representing the most popular targets. The hallmark of a pharmacological chaperone is its ability to bind a protein specifically and stabilize it. This property can be beneficial for curing diseases that are associated with protein mutants that are intrinsically active but unstable. The total activity of the affected proteins in the cell is lower than normal because they are cleared by the quality control system. Although most pharmacological chaperones are reversible competitive inhibitors or antagonists of their target proteins, the inhibitory activity is neither required nor desirable. This issue is well documented by specific examples among which those concerning Fabry disease. Direct specific binding is not the only mechanism by which small molecules can rescue mutant proteins in the cell. These drugs and the properly defined pharmacological chaperones can work together with different and possibly synergistic modes of action to revert a disease phenotype caused by an unstable protein.

## 1. Introduction

Pharmacological chaperones have ceased to be a niche category and have entered the clinical practice for some rare diseases caused primarily by protein instability. Many reviews exist to cover this subject with different points of view (a few examples [1,2,3,4,5,6,7,8,9,10,11,12,13,14,15]). We wanted to contribute by providing the readers with a list of research papers organized per disease that covers the years from 2000 to 2018. Different protein targets have been addressed, although enzymes associated with lysosomal storage disorders represent altogether half of the cases and most pharmacological chaperones that have been proposed are their reversible specific inhibitors. It is difficult to write a comprehensive report hence we chose to use the example of Fabry disease for which a drug has recently been approved, as a *leitmotiv* to put in evidence certain concepts that we believe require some clarification. We discussed a few papers to emphasize successes, stress limits, and indicate possible solutions.

Even in the most favorable cases, such as that of the drug approved for Fabry disease, inhibitors are not the ideal drugs as they can be able to stabilize their target proteins but might not be able to fully revert the disease phenotype. It has been proposed to modify first generation pharmacological chaperone to enhance their therapeutic effects.

Specifically, binding and stabilization of a protein target define a pharmacological chaperone, but a certain degree of confusion exists about the assignment of this term. Other small molecules that rescue mutant proteins in the cell without direct binding. Beyond the correctness of the definition, the possibility of employing different types of small molecules in synergy with pharmacological chaperones can potentiate their therapeutic effect.

## 2. Pharmacological Chaperones: The Time-Lapse

The term “pharmacological chaperone” was introduced by Morello and co-workers in 2000 [16] to define the action of a specific antagonist of the receptor of vasopressin. Missense mutations of the receptor cause Nephrogenic Diabetes Insipidus. If the antagonist is administered to cells carrying a mutant form of the receptor that is unable to reach the cell surface, it favors the accumulation of the mature protein. The authors wrote that molecules like the antagonist of vasopressin “would act as pharmacological chaperones that promote receptor processing through their specific binding activity” [16].

Specific binding is the hallmark that serves to distinguish pharmacological chaperones from other small molecules that can be useful in the treatment of diseases caused by unstable proteins. Since the pioneering work of Morello, the fortune of the term raised and the number of research articles mentioning “pharmacological chaperone” per year increased significantly. The approach with pharmacological chaperones was extended to other diseases.

**Figure 1:**
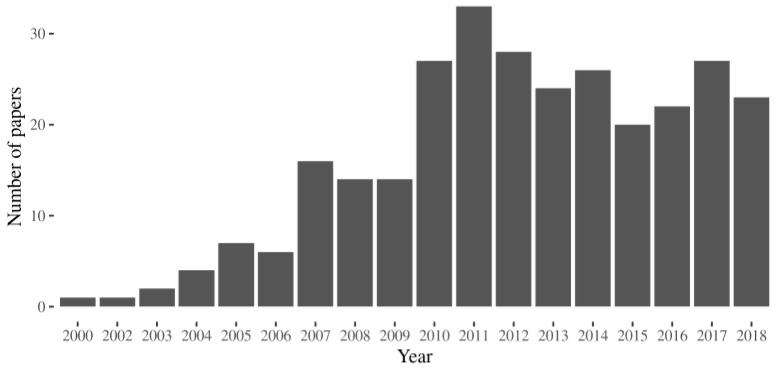
The number of the research articles indexed in Scopus that cite the term “pharmacological chaperone” in the title, in the abstract or the keywords and a specific disease.

We looked in UniProt [17] for the human proteins associated with MalaCards [18], the human disease database. We extracted the names of the diseases associated with each entry from the annotation in UniProt. The list (column 2 in Table S1) is partially redundant because we wanted to take into account that a given disease can be known with different names. For instance mutations affecting the Uniprot entry P10253 cause a disease known with different names, acid maltase deficiency, alpha-1,4-glucosidase deficiency, and, cardiomegalia glycogenica, gaa deficiency, glycogen storage disease, glycogenosis ii or Pompe disease. We queried Scopus to search for articles that contained the term “pharmacological chaperone” and one of the disease names extracted from Uniprot in either the title, the abstract or the keywords, respectively. In Figure 1 we provide a histogram describing the distribution of the research papers per year.

The association of the papers with specific target proteins required manual curation of data. Those papers for which the association with the protein target was not evident in the abstract were excluded from the analysis. For the others, we added the annotations of the protein target, i.e., the functional type, the localization (Table 1). 

## 3. Pharmacological Chaperones: Rational and Application

**Figure 2:**
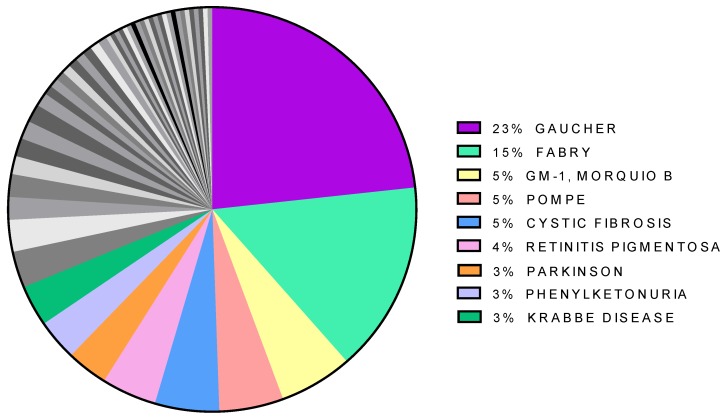
Distribution of papers citing “pharmacological chaperone” per disease. The less represented diseases are in gray.

Pharmacological chaperones have been tested for many diseases but the distribution of papers is not even. Approximately one-fourth of them concern Gaucher disease, and more than half the lysosomal storage disorders (LSD: Gaucher, Fabry, GM-1, Pompe) all together (Figure 2). Besides LSDs, the diseases most frequently associated with pharmacological chaperones are cystic fibrosis, retinitis pigmentosa, phenylketonuria, Parkinson.

**Figure 3:**
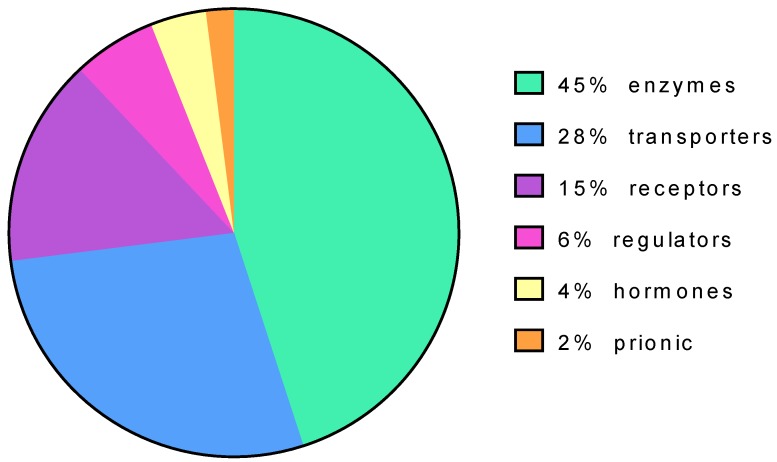
Protein targets of pharmacological chaperones based on their function.

Protein targets of pharmacological chaperone can be diverse. Enzymes, in particular transferases, represent the most populated group (45%) followed by transporters (28%) and receptors (15%) (Figure 3).

**Figure 4:**
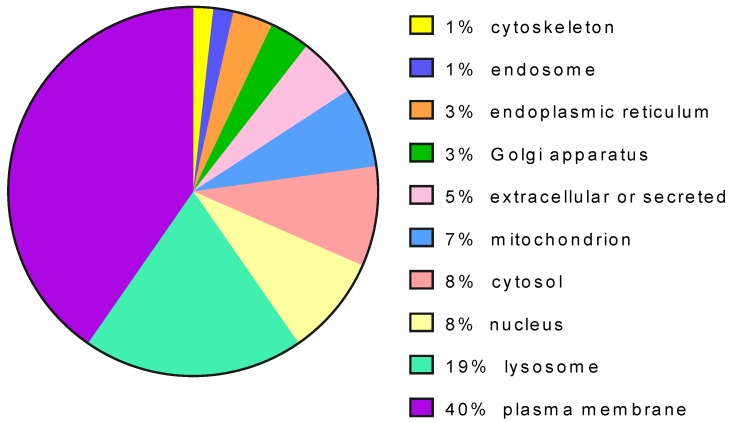
Distribution of protein targets of pharmacological chaperones per cell localization.

The proteins targeted by pharmacological chaperones can have different subcellular localization and, in the majority of the cases, are translocated across the endoplasmic reticulum (Figure 4).

## 4. Pharmacological Chaperones: Direct Specific Binding to Folded or Partially Folded Target Proteins as a Mechanistic Hallmark

Pharmacological chaperones are low molecular weight chemical molecules and should not be confused with molecular chaperones that are proteins [19,20]. According to the original definition by Morello and co-workers [16], pharmacological chaperones exert their action by binding specifically their target proteins. Direct specific binding differentiates pharmacological chaperones from other low molecular weight chemical molecules, such as chemical chaperones and regulators of protein homeostasis, that promote correct processing of a pathological mutant by different mechanisms.

In principle, direct binding and stabilization should be tested on purified mutants. This is unpractical for mutants that have disulfide bridges and post-translational modifications such as those that are secreted, that are resident in membranes (e.g., plasma membrane) or the endomembrane system (e.g., lysosomes, Golgi, endoplasmic reticulum or endosomes) because they cannot be produced and purified with a high yield. They represent the majority of the cases because proteins that transit in the endoplasmic reticulum are the most frequent targets of pharmacological chaperones (Figure 4).

**Figure 5:**
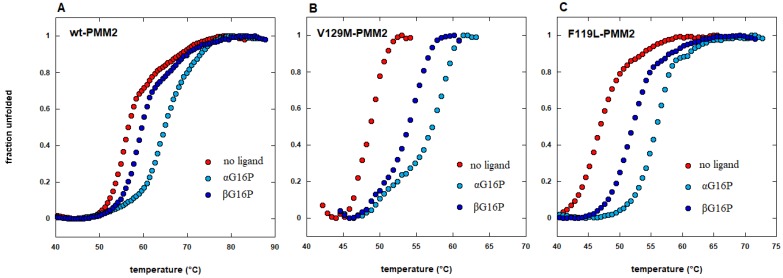
The effect of pharmacological chaperones is observed on responsive mutants as well as on wild type proteins. Thermal shift analysis of wild type phosphomannomutase2 (wt-PMM2 in panel A) and two pathological mutants (V129M-PMM2 in panel B and F119L-PMM2 in panel C) in the absence or in the presence of two different pharmacological chaperones, 1,6-alpha glucose-bisphosphate (αG16P) or 1,6-beta glucose-bisphosphate (βG16P) (extracted from reference [21]).

However, although the effect of pharmacological chaperones is more evident on responsive mutants, it can be observed on the corresponding wild type proteins too (an example is provided in [21] and is shown in Figure 5). This means that a preliminary screening can be run on the wild type protein.

Direct protein stabilization can be proved by chemical-physical techniques such as differential scanning calorimetry [22], by circular dichroism, i.e., measures of ellipticity as a function of temperature [23], or as a function of denaturant concentration [24]. Recently thermal shift assay has gained great popularity because it can process many samples at the same time; it requires small amounts of protein and equipment that is available in the majority of biomedical laboratories [25,26,27,28]. Incubation at a relatively high temperature of purified wild type protein targets in the presence or the absence of drugs followed by the measurement of residual enzymatic activity was used for the screening of relatively large libraries [29]. In some cases, the stabilizing effect of chaperones was tested in vitro on mutant proteins that were present in cellular extracts. Often the extracts were incubated in the absence or the presence of the chaperone at different temperatures and then assayed under standard conditions to measure the residual activity [30]. Alternatively, the extracts were incubated at different concentrations of urea, denatured proteins were selectively degraded by limited proteolysis and folded undigested proteins were detected by Western blot [24].

## 5. Pharmacological Chaperones: The Quick Path to Success is not Always the Best One

Most pharmacological chaperones have been discovered among reversible competitive inhibitors and have been tested for their stabilizing effect with the techniques mentioned beforehand. In this paper, we would like to stress on the prevalence of inhibitors among pharmacological chaperones occurred for practical reasons since many targets are enzymes (Figure 3). Competitive inhibitors often resemble chemically known substrates or products and an activity-based screening is straightforward. Indeed, competitive inhibitors are ligands that bind preferentially the folded state of an enzyme, and for this reason, they stabilize the protein. However, the stabilizing activity is required for chaperones, whereas the inhibitory activity is neither required nor desirable. Hence the concept of chaperone and that of inhibitor should not be assimilated.

The development of drugs for Fabry disease well illustrates this issue.

Fabry disease is an X-linked lysosomal storage disorder due to mutations in the gene *GLA* that encodes acid alpha-galactosidase A (AGAL). The enzyme cleaves globotriaosylceramide, generating galactose that is a product and a reversible inhibitor of the enzyme. There exist more than 400 pathological missense mutations, the majority of which reduce the stability of the protein but do not affect the active site [31,32]. In 1995, some years before Morello et al. had coined the term “pharmacological chaperone,” Okumya et al. [33] showed that galactose stabilizes missense mutants of alpha-galactosidase. They showed that administering 100 mM galactose to cells expressing certain missense mutants, a greater amount of the protein could be detected by Western blot and consequently a higher activity could be measured in the extracts. The authors explained the effect suggesting that the ligand stabilized responsive mutant AGAL. A few years later, Frustaci et al. [34] reported that infusions of galactose (1 g per kg body weight), improved the cardiac function of a Fabry patient. Interestingly the therapy was administered every other day. Although galactose cannot be considered a practicable therapeutic agent because of the high dosage needed to measure the effect in vitro and in vivo, the road was marked toward the usage of pharmacological chaperones for the treatment of Fabry patients carrying missense destabilizing mutations. In 1986 1,5-dideoxy-1,5-iminogalactitol, which is an iminosugar analog of galactose where the endocyclic oxygen is substituted by nitrogen, was synthesized [35,36]. The IUPAC name of that molecule is (2R,3S,4R,5S)-2-(Hydroxymethyl)-3,4,5-piperidinetriol, the usual name is 1-deoxygalactonojirimicin abbreviated in DGJ, but other names have also been employed, 1,5-dideoxy-1,5-imino-D-galactitol,1-deoxygalactonojirimycin, 1-deoxygalactostatin, Amigal, Migalastat, Galafold (trademark). DGJ was considered a good candidate as a practicable therapeutic agent because its *K*_i_ is at least two or three orders of magnitude lower than that of galactose [37]. In 1999 it was demonstrated that DGJ is more potent than galactose as a stabilizer of responsive AGAL mutants [30]. When DGJ was administered at 20 micromolar for four days to the R301Q or Q279E lymphoblasts from Fabry patients, it raised the residual activity in the cell lysates by seven–eight folds. Although Fan and co-workers showed that DGJ binds R301Q in vitro and correctly interpreted the mode of action of the drug, they proposed “the concept of ‘chemical chaperon’ to describe a small molecule whose function is to assist a protein to fold properly and enter normal processing pathway smoothly.” The term “chemical chaperon” for specific stabilizing ligands would have been soon overtaken by “pharmacological chaperone.”

Since the pioneering work by Fan and co-workers, the effect of DGJ has been tested on hundreds of missense mutations either on cells derived from patients or on transfected cells. To overcome the difficulty of obtaining lymphoblasts or fibroblasts from patients, Benjamin and co-workers [38] have developed a cell-based assay in HEK-293 cells that consents to test any mutations. After having been transiently transfected with expression vectors encoding *GLA* mutants, the HEK-293 cells are exposed to the drug, washed, and lysed. The amount of AGAL is quantified in the extracts by Western blot and standard enzymatic assays with a fluorogenic substrate. Almost 50% of the missense mutations tested so far are responsive to DGJ [39]. However, activity in cell extracts should not be confused with intracellular activity. AGAL dosage on cell extracts, in transfected cells as well as in cells derived from patients, represents a simple and effective way to demonstrate the stabilization of responsive mutants but does not prove that AGAL activity is increased in the presence of the drug in vivo.

DGJ is a potent inhibitor of AGAL, it binds and stabilizes the enzyme at the neutral pH as well as at acidic pH, (K_DpH7_ = 3.7 × 10^−9^ mol/L, K_DpH5_ = 6.0 × 10^−9^ mol/L) [37]. It is unlikely that DGJ stabilizes AGAL in ER without inhibiting it in the lysosomes. A proof of the double intracellular activity of the drug, the beneficial AGAL stabilization on one side and the deleterious AGAL inhibition on the other side, comes from the experiment carried out by Valenzano and co-workers [40]. They treated the Fabry fibroblasts carrying the responsive mutations R301Q or L300P with DGJ either with a discontinuous regimen, seven days followed by a three-day washout, or for a continuous regimen, ten days without washout. They observed augmented intracellular AGAL activity, namely the clearance of the AGAL substrate that accumulates in the cells of the patients, only when the treatment with the drug was discontinuous. The result was confirmed in a mouse model where a “4 on/3 off” regimen was more effective than a daily administration of DGJ to reduce the substrate of AGAL [41]. The benefits of an intermittent administration of DGJ can be explained by the fact that the half-life of most AGAL mutants is longer than the half-life of DGJ in vivo. The therapy would have two alternating phases. In the first one, i.e., in the presence of the drug, the enzyme would be stabilized but inhibited; in the second one, i.e., in the absence of the drug, the enzyme would be active but slowly degraded.

DGJ was approved by FDA for the use of responsive mutations [42]. An intermittent regimen is adopted in patients, and 150 mg of the drug is administered orally every other day.

The first reports on real clinical cases suggest that the beneficial long terms effects of DGJ should be evaluated carefully. In fact, a raise in AGAL residual activity has been observed in the leucocytes in patients of a Swiss cohort but regrettably, it did not correlate with a decrease in lyso-Gb3 concentration measured in dried blood spots [43].

## 6. Pharmacological Chaperones: Promising Drugs with a Restriction

Pharmacological chaperones cannot be used by all patients affected by a given genetic disease. They act by binding specifically unstable folded (or partially folded) mutant proteins. Hence they are not useful in all the cases, e.g., in which the protein is absent because the gene is affected by a deletion, a stop gain mutation, a splicing mutation, or a mutation occurring in the regulatory regions. Frameshift or stop loss could be treated if the mutation caused the production of a protein with some few extra amino acids at the carboxy-terminus that is able to fold. Pharmacological chaperones are of no use if the active site is affected. Hence, some mutations can be excluded *ab initio*, the other ones, which are potentially responsive, must be tested individually. The situation is even more complex in cases where patients are composite heterozygous and hetero-oligomeric proteins can be formed [44]. For some diseases the percentage of responsive mutations is low (for example Pompe disease [45]), for others, it is relatively high (for example Fabry disease [39]). For some diseases, there are prevalent mutations that can be prioritized in the analysis (for example cystic fibrosis [46,47] and Gaucher disease [48,49]). For other diseases, this not the case and all potentially responsive disease mutations must be systematically tested. In the last case, i.e., when there are a large number of private mutations, it is very important to have a cellular model for the test because it is unpractical to obtain cells from patients. Moreover, it is important that the test is carried out by independent labs in order to assure the robustness of the results. Preliminary tests are carried out assessing the ability of a pharmacological chaperone (and more in general of small molecules) to increase the intracellular concentration of the target protein. In the case of Fabry disease, an enzymatic assay is carried out on the extracts of transiently transfected cells [38]. This test can be carried out on any mutation; it is relatively simple and practical protocols are available [50]. Successive experiments should be carried out to assess the ability of the drug to revert the phenotype of the disease, in cell models or, more ambitiously, in animal models. DGJ has been approved for the therapy of responsive Fabry patients. Hence it is not surprising that for this disease a large number of potentially responsive genotypes have been tested by the company that developed the drug into a commercial product, galafold (trademark) [38,40,51] or by independent laboratories [50,52,53,54]. A database that keeps track of all results, FABRY_CEP, helps clinicians Choose Eligible Patients [55].

## 7. Pharmacological Chaperones: Improvement of a Drug

DGJ is able to stabilize a large proportion of missense mutations associated with Fabry disease and has been approved for the therapy and its long-term effects on the phenotype are still under verification. It might not be the ideal drug. However, certainly, it represents a good starting point. In the meantime, moving from the structure of DGJ, similar glycomimetic molecules have been proposed.

Its enantiomer L-DGJ is 1000-fold less active than D-DGJ and its mode of action is different because it is a non-competitive inhibitor of AGAL. Interestingly the two sugars act synergistically [56].

Iminosugars like DGJ are charged molecules with a negative octanol-water partition coefficient (Log Po/w) that indicates a low capacity to cross membranes [57]. The most obvious modification to enhance lipophilicity was alkylation. Unfortunately, N-alkylation, which was useful for molecules such as 1-Deoxynojirimycin, lowered DGJ efficacy [58].

Druglikeness could be improved synthesizing DGJ-thioureas derivatives. DGJ is an iminosugar analog of galactose. Galactose, in turn, is the terminal part of the natural substrate globotriaosylceramide. Hence it could be expected that extending the structure of DGJ it would be possible to derive a more selective and potent pharmacological chaperone. Although DGJ-thioureas derivatives inhibited AGAL at neutral and at acidic pH similarly to DGJ, the authors [59] stated that *p*-methoxyphenyl and the fluorophenyl thiourea derivatives had a chaperoning effect superior to that of DGJ in cells.

DGJ-thioureas can be described as compounds in which thiourea bridges two moieties, a sugar-like part, which in chemical terms is known the glycone to a non-sugar-like part, which in chemical terms is defined as a non-glycon. Ortiz Mellet and co-workers [60] proposed synthesizing a pH-sensitive thiourea. The non-glycone moiety is an orthoester group, which is stable at neutral pH but it is easily degraded at acidic pH. Hence the extended contacts with the target enzyme are possible in the endoplasmic reticulum but not in the lysosomes. When the DGJ-thiourea-orthoester PC was administered to fibroblasts derived from Fabry patients, it produced a reduction of Gb3 far more significant than that obtained with the parental drug DGJ. Remarkably, the approach can be extended in principle to all lysosomal diseases and a proof of concept was offered by the same authors who synthesized 1-deoxynojirimycin (DNJ)-thiourea-orthoesters for the cure of Gaucher disease.

pH-sensitive inhibitors can be found among molecules that do not look like substrates or products, screening large collections of small molecules. This was the case of ambroxol that is a mixed-type inhibitor and a stabilizer of lysosomal acid glucosylceramidase (Gaucher disease) and was found screening a library of 1040 FDA-approved compounds. It acts in the endoplasmic reticulum better than in the lysosome because its *K*_i_ is 5 times lower at pH 7 than at pH 5.6 and reduces the storage of the glucosylceramide in cells harboring a responsive mutation [29]. A similar reduction of glucosylceramide was not obtained with another inhibitor, the iminosugar isofagomine, which binds the enzyme at neutral pH as well as at acidic pH. Ambroxol can rescue some mutants of lysosomal alpha-galactosidase and alpha-glucosidase in cells harboring responsive mutations of Fabry or Pompe disease too, but in these cases, it is effective only if it is co-administered with the respective iminosugar pharmacological chaperones 1-deoxygalactonojirimycin (DGJ) and 1-deoxynojirimycin (DNJ) [61]. How comes that ambroxol is active on different enzymes? It might be a promiscuous ligand because lysosomal glycosidases, such as glucosylceramidase, alpha-galactosidase, and alpha-glucosidase share the same TIM-barrel architecture and have very similar structures [22]. On the other hand, ambroxol could stabilize glucosylceramidase not only by binding the protein but also by other mechanisms and these other mechanisms could be effective for other lysosomal storage disorders too.

Indeed most efforts to develop a pharmacological chaperone useful in clinical practice were concentrated on Gaucher disease (Figure 2). Isofagomine, an unmodified iminosugar, appeared to be very promising in stabilizing mutant glucosylceramidase [62] but did not reduce the accumulation of lipids significantly [14,63]. Besides the approaches already mentioned with alkylated or pH-sensitive molecules, another interesting option was evaluated. It consisted of the synthesis of compounds where nitrogen with high sp^2^-hybridation character takes the place of the amine-type endocyclic nitrogen found in iminosugars [64].

## 8. Pharmacological Chaperones: Besides the Inhibitors

Besides inhibitors, other ligands can act as pharmacological chaperones. It has been proposed for Fabry disease to look for allosteric ligands that stabilize missense unstable mutants but do not bind the active site and do not inhibit the activity. These molecules would be the ideal pharmacological chaperones. In a pilot study one molecule, 2,6-dithiopurine, was identified by in silico docking and tested by thermal shift assay before passing to tests on transfected cells [65]. Another example is offered by an elegant approach by Millet and co-workers [66] who were looking for drugs to cure congenital erythropoietic porphyria, which is caused by deficiencies of uroporphyrinogen III synthase. They used in silico docking to identify an allosteric binding site on the surface of the enzyme, they looked for ligands among 2500 diverse chemical fragments, they tested the binding of the best hits by thermal shift assay and NMR. The most promising chemical fragments were used to search a library of approved drugs and look for similar compounds. Millet and co-workers found that ciclopirox, an anti-fungal, could stabilize uroporphyrinogen III synthase in cells. This paper shows that drug repositioning should be always considered since this would cut the costs and the time needed to offer actual therapy to the patients [67]. Besides soluble enzyme multi-pass, G-coupled receptors have been targeted with allosteric modulators. Three molecules, cinacalcet, NPS-2143, and NPS-568 respectively, an FDA approved and two investigational drugs, were able to recover membrane expression and impaired function of some pathological mutants of the human calcium-sensing receptor [68,69]. The thienopyrimdine, Org 42599, a derivative of an allosteric modulator of the luteinising hormone receptor, was able to rescue several pathogenic mutants that are retained intracellularly and promote their localization on the plasma membrane and maturation. However, only some of the rescued mutants regained activity [70].

Besides allosteric non-inhibitory ligands, other types of molecules should be considered such as activators compounds. In this case, the example comes from PMM2-CDG a disorder caused by deficiencies in the gene encoding phosphomanno-mutase2. The enzyme catalyzes the conversion of mannose 6-phosphate into mannose 1-phosphate and uses as activator glucose 1,6 bisphosphate. The activator binds the active site but is not consumed. It stabilizes several pathological mutants [44,71,72]. Other non-inhibitory ligands, which bind the active site and stabilize some mutants in the cells, have been described, 11-cis-retinal and tetrahydrobiopterin, which are the natural cofactors of rhodopsin [73] and phenylalanine-hydroxylase respectively, for retinitis pigmentosa and phenylketonuria [74,75,76].

## 9. Pharmacological and Chemical Chaperones: Two Types of Drugs Often Confused

**Figure 6:**
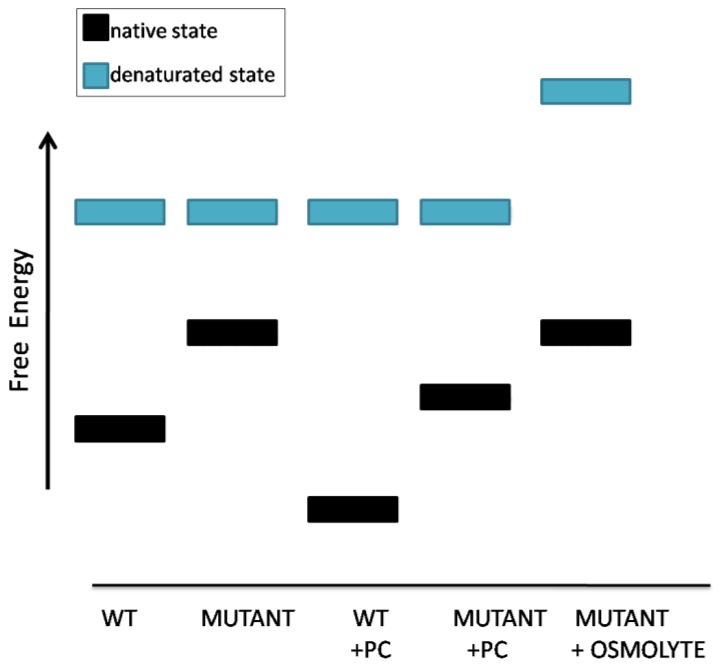
Schematic representation of the effects of pharmacological chaperones or osmolyte chemical chaperones on protein stability.

So far we have described pharmacological chaperones. We now move to other small molecules, the so-called “chemical chaperones.” They have been proposed for the treatment of genetic diseases that are caused by unstable proteins too, but “chemical chaperones,” such as osmolites and hydrophobic compounds, do not bind their targets specifically. It has been proposed that osmolites such as betaine, sarcosine, glycerol, glycine, trimethylamine N-oxide increase the energy of the unfolded state of proteins. Hence pharmacological chaperones would raise the difference between the free energy of the folded and unfolded states by lowering the first one whereas osmolites would obtain the same effect mainly by raising the second one [2] (Figure 6).

Osmolytes are relatively aspecific and were proved active on different proteins such as cystathionine *β*-synthase [77], aspartylglucosaminidase [78], adenosine triphosphate (ATP)-binding cassette subfamily A member 3 [79], alpha-1 anti-trypsin [80] in cellular models. Particularly interesting is the example offered by another chemical chaperone, 4-phenyl-butyrate (4-PBA). 4-PBA is a drug approved for the treatment of disorders of the urea cycle. Besides being an ammonia scavenger, it can stabilize a wide range of proteins. In fact, 4-PBA contains an aromatic moiety and is able to bind hydrophobic patches of unfolded or partially folded proteins. For this reason, it acts as a “hydrophobic chaperone.” It can rescue DeltaF508 cystic fibrosis transmembrane conductance regulator (CFTR) [81], cystathionine β-synthase [77], alpha-1 anti-trypsin [80], ABCC6 protein [82], ATP-binding cassette transporter A1 [83], parathyroid hormone [84], and Wilson protein (Copper-transporting P-type ATPase ATP7B) [85] and the list might not be exhaustive. However, the interaction with hydrophobic patches of unstable proteins can only in part account for the effects of 4-PBA. In fact, it down-regulates the expression of Hsc70 [86], transiently increases Hsp70 expression [87], reduces the upregulation of ER stress markers CHOP and GRP78 [88], and inhibits histone deacetylases activity [89,90] and signaling [87]. Disentangling the different roles of 4-PBA can be difficult [91].

## 10. Other “Small Molecules”: Alternative or Synergistic Approaches to Treat Diseases Caused by Destabilizing Missense Mutations

Small molecules can stabilize missense mutants acting indirectly. This is the case of heat-shock protein inducers. The first example is provided by 4-PBA that could act as a hydrophobic chaperone as well as an inducer of hsp70. Other heat-shock response co-inducers have been described. Arimoclomol is an aromatic heteromonocyclic compound that underwent clinical trial phase 3 for Amyotrophic Lateral Sclerosis [92]. It rescues glucocerebrosidase in fibroblasts of Gaucher patients both in terms of quantity and in terms of maturation [93] and prevents aggregation of a missense mutant of rhodopsin, P23H, which is frequently encountered in retinitis pigmentosa [94]. Kelly et al. [95] suggested that co-administration of a proteostasis regulator and of pharmacological chaperone could have a synergistic effect on rescuing missense pathogenic mutants. They proved their concept utilizing Celastrol, a triterpene derived from the traditional Chinese herb known as Thunder of God Vine and an alkylated deoxynojirimycin. The first molecule activates the heat shock protein response in various cell types [96] and inhibits proteasome [97], while the latter binds and stabilizes specific mutants of lysosomal acid glucosylceramidase which are responsible for Gaucher disease [98]. A similar approach with proteostasis regulators was tested with Fabry disease [99].

## 11. Conclusions

**Figure 7:**
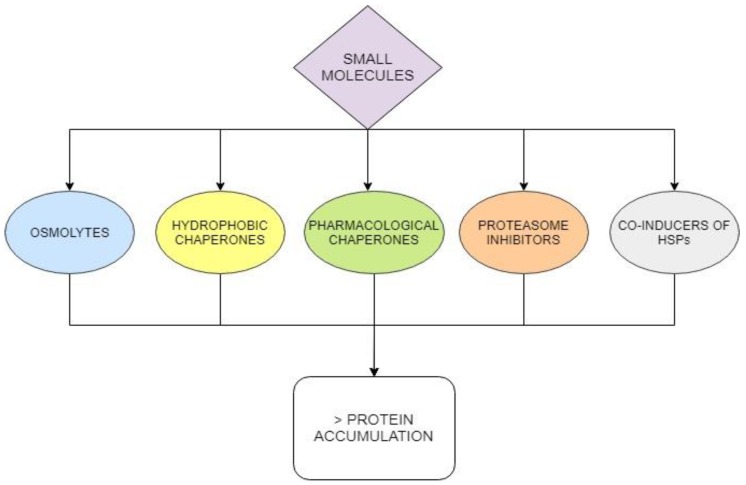
Different classes of small molecules can rescue proteins destabilized by mutations and increase their intracellular concentration.

Most disease mutations destabilize the affected protein but do not affect the active site and mutant pathogenic proteins can be rescued. This goal can be achieved using small molecular weight molecules that can be administered orally and reach difficult districts such as the central nervous system. The approach is not limited to enzymopathies and has been transversally proposed for many diseases. Admittedly the pathway from tests in vitro to a clinical trial is long but the steps to be followed are clear since they are well established in traditional medicinal chemistry. On the contrary, other very promising approaches such as gene therapy are still at their infancy. Different classes of small molecules (Figure 7) can be considered.

Pharmacological chaperones play a special role among them. In fact, they are by far more specific than the others for a given protein. It is important to realize that most pharmacological chaperones tested so far are inhibitors or antagonists of their targets and that this occurred only for practical reasons. Special regimens must be envisaged to maximize their stabilizing activity and minimize their inhibitory activity. We might consider these molecules as first-generation drugs. They played an important role to prove the value of the approach but more effective molecules should be sought. If this was not feasible, we should consider combined therapies, using a specific pharmacological chaperone at a low dosage but potentiated by other types of small molecules. Unspecific drugs that promote protein folding and restore proteostasis can be useful for diverse diseases and can be found among approved or experimental drugs in the first place.

## Figures and Tables

**Table 1:** Distribution of research papers per disease and per protein target. The table summarizes how many articles use the term “pharmacological chaperone” and the corresponding disease context (we chose arbitrarily what we considered the most representative name of the disease). The UniProt entry of the affected protein, the name of the gene, the protein type, and the protein localization are also shown.

Disease	Gene	Uniprot Code	Protein Type	Subcellular Location	N. Of Articles
Gaucher	*GBA*	P04062	enzyme	lysosome	64
Fabry	*GLA*	P06280	enzyme	lysosome	42
GM-1, Morqio B	*GLB1*	P16278	enzyme	lysosome	16
Pompe	*GAA*	P10253	enzyme	lysosome	14
Cystic fibrosis	*CFTR*	P13569	transporter	plasma membrane	14
Retinitis pigmentosa	*RHO*	P08100	receptor	plasma membrane	12
Phenylketonuria	*PAH*	P00439	enzyme	cytosol	9
Krabbe disease	*GALC*	P54803	enzyme	lysosome	9
Nephrogenic diabetes insipidus	*V2R*	P30518	receptor	plasma membrane	8
Long QT Syndrome	*KCNH2*	Q12809	transporter	plasma membrane	7
Parkinson	*PARK7*	Q99497	enzyme	plasma membrane, nucleus, mitochondrion	5
Niemann-Pick	*NPC1*	O15118	receptor	lysosome	5
Hyperoxaluria	*AGXT*	Q86XE5	enzyme	mitochondrion	5
Obesity	*MC4R*	P32245	receptor	plasma membrane	4
GM-2, Sanfilippo syndrome	*GNRHR*	P07686	enzyme	lysosome	4
GM-2, Tay-Sachs syndrome	*HEXB*	P06865	enzyme	lysosome	4
Galactosemia	*HEXA*	P07902	enzyme	cytosol	4
Hypoparathyroidism	*PTH*	P01270	hormone	extracellular or secreted	3
Parkinson	*GALT*	P04062	enzyme	lysosome	3
Hypogonadotropic hypogonadism	*ATP7B*	P30968	receptor	plasma membrane	2
Wilson	*PMM2*	P35670	transporter	Golgi apparatus	2
PMM2-CDG	*SLC26A4*	O15305	enzyme	cytosol	2
Pendred	*MMAB*	O43511	transporter	plasma membrane	2
Methylmalonic aciduria	*ABCB4*	Q96EY8	enzyme	mitochondrion	2
Intrahepatic cholestasis	*DRD4*	P21439	transporter	plasma membrane	2
Hyperactivity disorder	*ABCC8*	P21917	receptor	plasma membrane	2
Diabetes	*GPR56*	Q09428	receptor	plasma membrane	2
Polymicrogyria	*PGK1*	Q9Y653	receptor	plasma membrane, extracellular or secreted	1
Phosphoglycerate kinase 1 deficiency	*SNCA*	P00558	enzyme	cytosol	1
Parkinson	*SUMF1*	P37840	regulator	presynaptic vesicle	1
Multiple sulfatase deficiency	*NPM*	Q8NBK3	enzyme	E.R.	1
Leukemia	*PKR2*	P06748	regulator	nucleus, cytoskeleton	1
Intrahepatic cholestasis	*ABCB11*	O95342	transporter	plasma membrane	1
Nocturnal frontal lobe epilepsy	*CHRNB2/CHRNA4*	P17787/P43681	transporter	plasma membrane	1
Hypomagnesemia	*CLDN16*	Q9Y5I7	transporter	plasma membrane	1
Creutzfeld-Jacob, Kuru	*PRNP*	P04156	unclear/prion	plasma membrane	1
Homocystinuria	*CBS*	P35520	enzyme	nucleus	1
Fibrodysplasia ossificans	*ACVR1*	Q04771	enzyme	plasma membrane	1
Epilepsy, Migraine	*SCN1A*	P35498	transporter	plasma membrane	1
Dystonia	*SLC2A1*	P11166	transporter	plasma membrane	1
Diarrhea (cholera toxin)	*NHE3*	P48764	transporter	plasma membrane	1
Diabetes	*KCNJ11*	Q14654	transporter	plasma membrane	1
Intrahepatic cholestasis	*ATP8B1*	O43520	transporter	plasma membrane, Golgi apparatus, E.R.	1
Breast cancer	*NBS*	O60934	regulator	nucleus	1
Amyotrophic lateral sclerosis	*SOD1*	P00441	enzyme	nucleus, mitochondrion	1
Amyloidosis	*VPS29/VPS26*	Q9UBQ0/O75436	transporter	endosome	1
Allan-Herndon-Dudley	*SLC16A2*	P36021	transporter	plasma membrane	1
Alkaptonuria	*HGD*	Q93099	enzyme	cytosol	1
Aspartylglucosaminuria	*AGA*	P20933	enzyme	lysosome	1
Ceroid lipofuscinosis	*PPT1*	P50897	enzyme	lysosome	1
Schindler disease	*NAGA*	P17050	enzyme	lysosome	1
Diabetes mellitus	*IAPP*	P10997	hormone	extracellular or secreted	1
GM-1	*IDS*	P22304	enzyme	lysosome	1
Morquio A, Hunter disease	*GALNS*	P34059	enzyme	lysosome	1

## Supplementary Materials

Supplementary materials can be found at https://www.mdpi.com/1422-0067/21/2/489/s1. Research papers citing “pharmacological chaperone” per disease Table S1.
